# Identification of an altered peptide ligand based on the endogenously presented, rheumatoid arthritis-associated, human cartilage glycoprotein-39(263–275) epitope: an MHC anchor variant peptide for immune modulation

**DOI:** 10.1186/ar2269

**Published:** 2007-07-23

**Authors:** Annemieke MH Boots, Henk Hubers, Milou Kouwijzer, Leontien den Hoed-van Zandbrink, Bernice M Westrek-Esselink, Cindy van Doorn, Rachel Stenger, Ebo S Bos, Marie-jose C van Lierop, Gijs F Verheijden, Cornelis M Timmers, Catharina J van Staveren

**Affiliations:** 1 NV Organon, Research Laboratories, Oss, The Netherlands

## Abstract

We sought to identify an altered peptide ligand (APL) based on the endogenously expressed synovial auto-epitope of human cartilage glycoprotein-39 (HC gp-39) for modulation of cognate, HLA-DR4-restricted T cells. For this purpose we employed a panel of well-characterized T cell hybridomas generated from HC gp-39-immunized HLA-DR4 transgenic mice. The hybridomas all respond to the HC gp-39(263–275) epitope when bound to HLA-DR4(B1*0401) but differ in their fine specificities. First, the major histocompatibility complex (MHC) and T-cell receptor (TCR) contact residues were identified by analysis of single site substituted analogue peptides for HLA-DR4 binding and cognate T cell recognition using both T hybridomas and polyclonal T cells from peptide-immunized HLA-DR4 transgenic mice. Analysis of single site substituted APL by cognate T cells led to identification of Phe265 as the dominant MHC anchor. The amino acids Ala268, Ser269, Glu271 and Thr272 constituted the major TCR contact residues, as substitution at these positions did not affect HLA-DR4(B1*0401) binding but abrogated T cell responses. A structural model for visualisation of TCR recognition was derived. Second, a set of non-classical APLs, modified at the MHC key anchor position but with unaltered TCR contacts, was developed. When these APLs were analysed, a partial TCR agonist was identified and found to modulate the HC gp-39(263–275)-specific, pro-inflammatory response in HLA-DR4 transgenic mice. We identified a non-classical APL by modification of the p1 MHC anchor in a synovial auto-epitope. This APL may qualify for rheumatoid arthritis immunotherapy.

## Introduction

In rheumatoid arthritis (RA), articular cartilage is destroyed by chronic inflammation that is characterised by activated lymphocytes and major histocompatibility complex (MHC) class II expressing cells in synovial tissue. The latter suggests the ongoing of an antigen-driven response [1]. Although the nature of the antigens responsible for RA pathogenesis is unknown, there is evidence that the disease-associated HLA-DR (B1*0401, 0404, 0405 and 0101) molecules are involved in disease pathogenesis [2]. The clinical observation that cartilage is likely to sustain the inflammatory response suggested a role for cartilage proteins as target autoantigens [3-7].

The human cartilage glycoprotein-39 (HC gp-39) is a 42 kDa glycoprotein with structural homology to the bacterial chitinase protein family [8-11]. Although its physiological function is unknown, its expression pattern suggests a role in tissue remodelling [9,12]. The case for an involvement of HC gp-39 in RA has been well-documented. Serum and synovial fluid HC gp-39 levels are elevated in inflammatory diseases and correlate with disease activity in RA [13,14]. HC gp-39 mRNA has been detected in synovial specimens and cartilage of RA patients but not in normal cartilage [9,15]. Also, an increased presence of HC gp-39-expressing monocytic cells in RA synovial tissue is correlated with the degree of joint destruction [16]. Furthermore, HC gp-39-derived peptides with good relative affinity for the RA-associated HLA-DR molecules were identified as dominant T cell epitopes in HC gp-39-immunized, HLA-DR4 transgenic mice [17]. Similarly, these T cell epitopes were recognized by peripheral blood mononuclear cells from RA patients [7,17,18]. Interestingly, peripheral blood mononuclear cells from RA patients were found to respond to HC gp-39 in a pro-inflammatory mode whereas cells from healthy donors responded in an anti-inflammatory mode [19]. The demonstration that endogenous presentation of the 263–275 dominant epitope in the context of the RA-associated HLA-DR molecules by synovial dendritic cells is specific for RA pathology adds to the relevance of this epitope for the disease process [20,21]. Recently, these findings were extended by the demonstration that antigen-presenting cells (APCs) in RA synovial fluid present endogenously processed HC gp-39-derived epitopes to T cells [22,23]. These observations combined imply that immunotherapeutic strategies based on the endogenous sequence may be beneficial for RA. Given the role of HC gp-39, the tolerizing potential of HC gp-39 in experimental arthritis [7,24] and the mere fact that a dominant epitope is endogenously expressed at the site of chronic inflammation, we sought to exploit the HC gp-39-derived epitope for antigen-based forms of therapy.

Cognate peptides may be modified at key T-cell receptor (TCR) recognition residues to create altered peptide ligands (APL). TCR interaction with APLs was found to alter the extent of antigen-specific T cell activation by skewing the cytokine profile of responding T cells or by anergy induction [25]. More importantly, in experimental models of autoimmune disease, APLs have been used successfully to deviate or dampen the pathogenic response [26-29]. The translation of this preclinical work into successful clinical trials, however, has proven difficult. In clinical trials on multiple sclerosis, adverse hypersensitivity reactions were seen with classic APLs modified at key TCR contact sites [30,31]. Current insights favour the notion that APLs based on MHC anchor substitutions may function as partial TCR agonists on the one hand and prevent unwanted immune reactivity on the other [32,33]. This approach may thus provide an improved option for APL therapy.

The objectives of the present study were first to identify the MHC and TCR contact residues in the RA-associated epitope using a panel of well-characterized specific T cell hybridomas, second to derive a molecular model to guide APL design, and third, to identify an APL capable of modulating the pro-inflammatory response to the synovial auto-epitope.

## Materials and methods

### Peptides and altered peptide ligands

**Figure 1:**
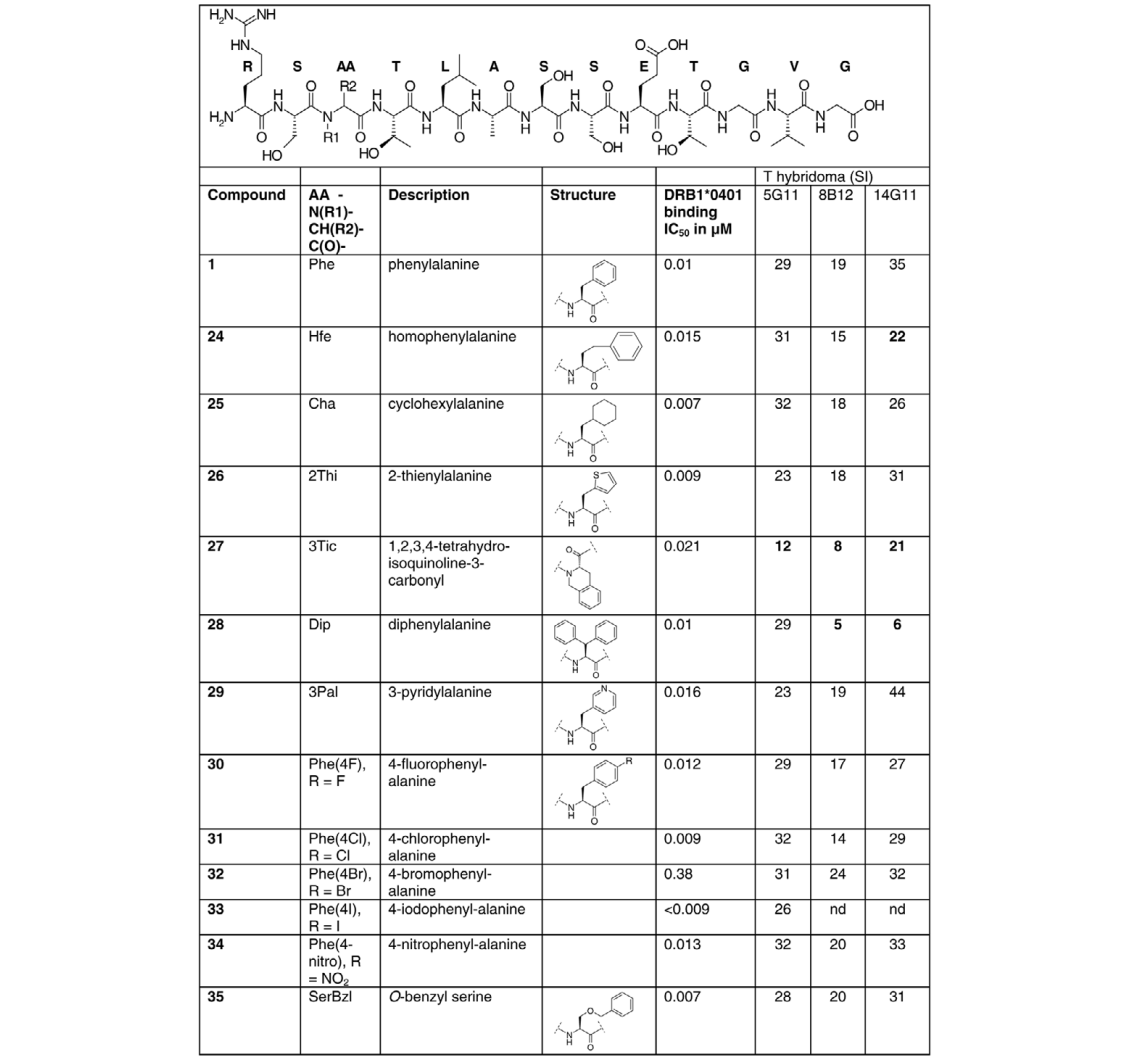
Structures of the HC gp-39(263–275) sequence (compound 1) and of a series of modified peptides at anchor position 1 (P1, Phe265; compounds 24 to 35) and associated bioactivities. Binding of MHC anchor variant peptides to HLA-DR4(B1*0401) was determined in a competition binding assay. IC_50 _= 50% inhibitory concentration. All peptides were found to bind HLA-DR4 with high relative affinity (IC_50 _ranged between 0.001 and 0.38 μM). The hybridoma response (IL-2 production) to wild-type (WT) peptide and MHC anchor variant peptides presented by HLA-DRB1*0401 expressing B lymphoblastoid cells as source of antigen-presenting cells is expressed as stimulation index (SI). The SI values are based on mean fluorescence counts derived from duplicate or triplicate measurements and calculated as the ratio of mean fluorescence counts of antigen stimulated cultures and control cultures. Background (no peptide added) values for hybridoma 5G11, 8B12 and 14G11 were 29,203, 16,288 and 7,152 mean fluorescence units/counts, respectively. The standard deviation of measurements did not exceed 15%. Values greater or less than 30% (2 × the standard deviation) of the positive control (response to WT peptide) are defined as super agonists (+30%) and partial agonists (-30%) respectively and are indicated in bold.

Peptides were synthesised by solid phase peptide synthesis. Purity and identity of the peptides were assessed by reverse phase high performance liquid chromatography and fast atom bombardment mass spectrometry, respectively. The fine epitope specificity of individual T hybridomas was determined using amino- and carboxy-terminally truncated peptides (1 to 9 in Table 1). In order to identify the residues interacting with MHC and/or the TCR, 12 single alanine substituted APLs and one aspartic acid substituted APL were synthesized (compounds 10 to 23 in Table 2) and tested. Finally, the phenylalanine residue at anchor position 1 (Phe265) was replaced by a series of well-known structural analogues (non-natural amino acids; 12 different compounds (24 to 35 in Figure 1)). With these modifications the amino acid side chain at position 265 was changed compared to that of the native peptide 1, whereas the peptide backbone remained unaltered. Only in the case of the more rigid bicyclic amino acid 3Tic (compound 27), was the peptide backbone also conformationally changed.

**Table 1:** Hybridoma response to amino- and carboxy-terminal truncated peptides of HC gp-39(263–275)

				Hybridoma response (SI)			
				
Compound	HC gp-39	Sequence	HLA-DR binding IC_50 _(μM)	5G11	8B12	14G11	20H5
				Vβ10b	Vβ10b	Vβ6	Vβ10b
1	263–275	RSFTLASSETGVG	0.12	**13**	**9**	**15**	**26**
2	263–274	RSFTLASSETGV	0.09	**7**	**8**	**10**	**22**
3	263–273	RSFTLASSETG	0.20	2	**5**	**5**	**15**
4	263–272	RSFTLASSET	7.0	1	1	1	2
5	264–275	SFTLASSETGVG	0.09	**12**	**10**	**15**	**27**
6	265–275	FTLASSETGVG	0.11	**11**	**6**	**8**	**24**
7	266–275	TLASSETGVG	>100	1	1	1	**5**
8	267–275	LASSETGVG	>100	1	1	1	1
9	264–274	SFTLASSETGV	0.12	ND	**7**	**13**	**22**

Binding of peptides to HLA-DR4(B1*0401) was determined in a competition binding assay. IC_50 _= 50% inhibitory concentration. IC_50 _< 1 μM = high affinity binder; IC_50 _between 1 and 10 μM = good binder; IC_50 _between 10 and 100 μM = intermediate binder; IC_50 _between 100 and 1,000 μM = poor binder. The decimals do not reflect the accuracy of the data but represent the calculated geometric means obtained in two experiments. The HLA-DRB1*0401 expressing B lymphoblastoid cell line was used as a source of antigen-presenting cells. Background values for hybridomas 5G11, 8B12, 14G11 and 20H5 were 8,147, 7,470, 8,598 and 35,749 mean fluorescence units/counts, respectively. Hybridoma IL-2 response is expressed as stimulation index (SI) calculated as the ratio of mean fluorescence counts of antigen-stimulated cultures and control cultures. Values in bold are considered positive (SI > 2). Data are representative of at least three independent experiments. ND, not determined.

**Table 2:** T hybridoma response to single site (alanine or aspartic acid) substituted altered peptide ligands of HC gp-39(263–275)

				Hybridoma response (SI)			
				
Compound	HC gp-39	Sequence	HLA-DR binding IC_50 _(μM)	5G11	8B12	14G11	20H5
1	263–275	RSFTLASSETGVG	0.08	**17**	**39**	**30**	**5**
10	R263A1	**A**SFTLASSETGVG	0.10	**23**	**42**	**28**	**5**
11	S264A2	R**A**FTLASSETGVG	0.10	**21**	**43**	**32**	**5**
12	F265A3	RS**A**TLASSETGVG	>35	2	**6**	2	**4**
13	T266A4	RSF**A**LASSETGVG	0.06	**7**	**14**	1	**5**
14	L267A5	RSFT**A**ASSETGVG	0.08	**4**	1	1	**4**
15	A268D6	RSFTL**D**SSETGVG	0.24	1	1	1	2
16	S269A7	RSFTLA**A**SETGVG	0.05	2	1	1	2
17	S270A8	RSFTLAS**A**ETGVG	0.05	**21**	**29**	**27**	**5**
18	E271A9	RSFTLASS**A**TGVG	0.04	1	1	1	2
19	T272A10	RSFTLASSE**A**GVG	0.04	1	**4**	1	1
20	G273A11	RSFTLASSET**A**VG	0.05	**7**	**32**	**6**	**5**
21	V274A12	RSFTLASSETG**A**G	0.06	**26**	**40**	**30**	**5**
22	G275A13	RSFTLASSETGV**A**	0.05	**17**	**30**	**20**	**5**
23	A3/A8	RS**A**TLAS**A**ETGVG	>36	ND	ND	ND	ND

Binding of peptides to HLA-DR4 (B1*0401) was determined in a competition binding assay. IC_50 _= 50% inhibitory concentration. IC_50 _< 1 μM = high affinity binder; IC_50 _between 1 and 10 μM = good binder; IC_50 _between 10 and 100 μM = intermediate binder. The HLA-DRB1*0401 expressing B lymphoblastoid cell line was used as a source of antigen-presenting cells. Background values for hybridomas 5G11, 8B12, 14G11 and 20H5 were 17,238, 8,773, 10,131 and 290,666 mean fluorescence units/counts, respectively. The standard deviation of measurements did not exceed 15%. Hybridoma IL-2 response is expressed as stimulation index (SI) calculated as the ratio of mean fluorescence counts of antigen stimulated cultures and control cultures. Values in bold are considered positive (SI > 2). Data are representative of at least three independent experiments. ND, not determined.

### MHC peptide binding assay

The binding of synthetic peptides to affinity-purified HLA-DR4 molecules was determined relative to a biotinylated marker peptide (HA307-319 (Y309F)) in competition binding assays as described [34]. In brief, a mixture of a fixed concentration of affinity purified HLA-DR4 (30 nM), biotinylated marker peptide (50 nM) HA309F, and increasing concentrations of competitor peptide (compounds 1 to 35) was incubated in binding buffer (pH 5) for 48 h at room temperature. HLA-DR bound marker peptide was separated from free marker peptide and the amount of bound marker peptide was detected and quantified by enhanced chemoluminescense. Inhibition curves were generated and the IC_50 _was determined. The concentration of competitor peptide inducing 50% inhibition of marker peptide binding was determined, and expressed as the 50% inhibitory concentration (IC_50 _in μM).

### Generation and characterization of T cell hybridomas from HLA-DR4(B1*0401) transgenic mice

The T cell hybridomas, provided by G Sonderstrup (Stanford, CA, USA), were generated as described [17]. In brief, human CD4, HLA-DRA*101/HLA-DRB*0401 triple transgenic (HLA-DR4trg) mice (devoid of I-Aβ) were immunized in the footpad with 50 μg of purified HC gp-39 mixed in incomplete Freund's adjuvant (IFA). Draining lymph nodes were isolated and T-cell hybridomas were generated by polyethylene glycol fusion of primed and *in vitro *antigen-restimulated lymph node cells with the AKR BW5147 fusion partner. Following fusion, growth-positive hybridoma wells were screened for reactivity to HC gp-39 and a set of 55 peptides (16 amino acids in length) covering the entire HC gp-39 sequence. HLA-DR4(B1*0401)-restricted hybridomas responsive to both the HC gp-39 protein and the immunodominant epitope 263–275 of HC gp-39 (for example, 5G11, 8B12, 14G11 and 20H5) were cloned, subcloned, characterised and selected for further study.

TCR Vβ usage by the T hybridoma clones was determined by staining of 5 × 10^5 ^hybridoma cells with monoclonal antibodies directed against 17 different mouse T cell receptors (mouse Vβ TCR screening panel, BD Pharmingen, catalogue no. 557004, San Jose, CA, USA). Samples were analysed using a flow cytometer (FACScan BD).

**Figure 2:**
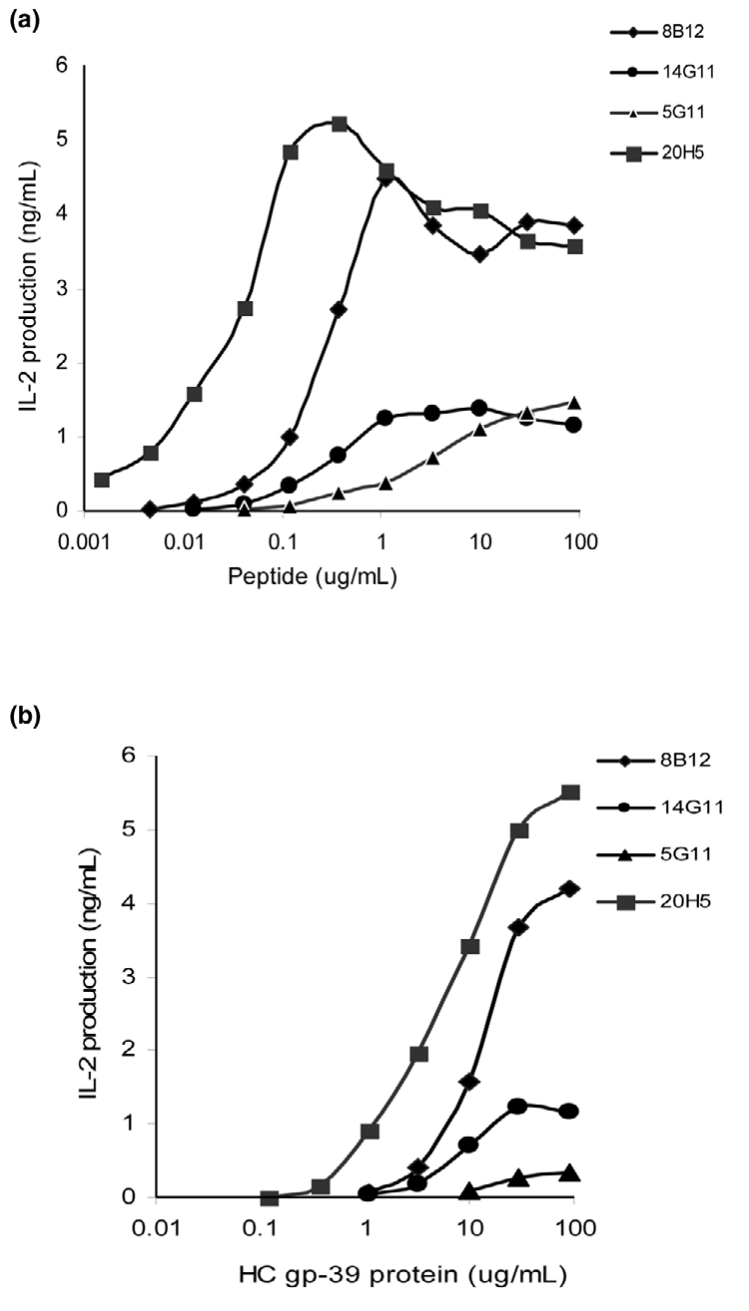
Dose response relationships for the four different hybridomas used in this study. **(a) **IL-2 production in ng/ml measured following stimulation with increasing concentrations of wild-type peptide. **(b) **IL-2 production in ng/ml measured following stimulation with increasing concentrations of HC gp-39 protein.

HC gp-39-specific IL-2 production by T hybridomas was measured in wells of round-bottomed microtiter plates. Hybridoma cells (2 to 5 × 10^4^) and irradiated APC (0.5 to 1 × 10^5^; 12,000 to 16,000 RAD) from the B lymphoblastoid cell line (BLCL) carrying the DRB1*0401 specificity were incubated in 150 μl volumes. Antigen was added in 50 μl volumes to duplicate wells. After 48 h, 100 μl of the culture supernatant was assayed for IL-2 production using a sandwich ELISA with Pharmingen antibodies specific for mouse IL-2. The antigen sensitivity of the hybridomas to both peptide and protein was determined (Figure 2). Half maximal stimulation was seen at wild-type (WT) peptide concentrations of 0.02 μg/ml for 20H5, 0.21 μg/ml for 8B12, 0.26 μg/ml for 14G11 and 2 μg/ml for 5G11 (Figure 2a). In later assays, an excess of peptide or APL (10 μg/ml), inducing maximal responses in all hybridomas, was tested.

### Molecular modelling of the HLA-DR4(B1*0401)/HC gp-39(263–275) complex

We extracted 13 unique crystal structures of TCR-MHC-peptide complexes from the Protein Data Bank [35]. Ten contained a MHC class I molecule and three a class II [36-38]. The crystal structure of HLA-DRA*0101/HLA-DRB*0401 with an influenza hemagglutinin peptide and the HA 1.7 TCR [38] was used as a template for a structural model, excluding the TCR. WHAT IF [39] was used to simply replace the peptide residues with the HC gp-39(263–275) residues. Using CHARMm (Accelrys Inc, San Diego, CA, USA), an initial energy minimization was performed, followed by a 10 ps molecular dynamics simulation to heat the system to 400 K and a 100 ps run at 400 K. Only atoms within 8 Å of the peptide were allowed to move during the molecular dynamics simulations. The atomic coordinates were saved to disk every picosecond, and after the simulation the stored 100 structures were all energy minimized to remove effects due to the high temperature. This procedure [40] leads to a favourable structural model and to an estimation of the binding strength via the average interaction energy between peptide and MHC molecule. Other alignments of the HC gp-39 peptide in the binding groove were also considered, and the alanine substituted peptides (Table 2) were modelled for validation purposes.

### Immunization of HLA-DR4trg mice with WT peptide or APL

Mice, obtained from L Fugger (Oxford, UK), were immunized in the hind footpads with 2 × 50 μg of purified recombinant HC gp-39, with 2 × 100 μg of the WT peptide or with 2 × 100 μg of the APL (F265(Dip); compound 28; Figure 1) mixed in IFA. Seven days later, popliteal lymph node cell suspensions (1 to 2 × 105 cells/well) were stimulated *in vitro *with HC gp-39 (50, 10 or 2 μg/ml), HC gp-39(263–275) or APL (10, 2 and 0.4 μg/ml). At 96 h, 50 μl of culture medium was harvested for analysis of antigen-specific cytokine production using Luminex technology (mouse cytokine 18 Plex Panel, catalogue no. 171-F11181, Biorad, Bio-Rad Laboratories, Hercules, CA, USA). In addition, cultures were labelled with tritiated thymidine (for 18 h) and antigen-specific proliferation was determined. We state that all animal experimentation received ethics approval and animals were treated according to the recognized guidelines.

## Results

### Hybridoma characterization: TCR Vβ usage and core epitope mapping

Hybridoma 14G11 was found to bear TCR Vβ6. All other hybridomas (5G11, 8B12 and 20H5) were found to carry Vβ10b (data not shown; Table 1). In order to define the minimal epitope region recognized by individual hybridomas, truncated peptides were assayed for HLA-DR binding and T cell recognition (Table 1). In line with previous studies [17], the binding data show that Phe265 is important for anchoring the peptide in the HLA-DR pocket; all peptides truncated beyond Phe265 (266–275 and 267–275) showed strongly reduced affinities (IC_50 _values > 100 μM). The 5G11 response was not affected by the amino-terminal Arg263 and Ser264. In agreement with the binding data, Phe265 is essential for 5G11 recognition. The carboxy-terminal truncations indicated that Val274 at the penultimate position of the peptide is essential for recognition. Thus, the minimal epitope recognized by 5G11 corresponds to amino acids 265–274. Recognition by the 8B12 and the 14G11 hybridomas centred around the 265–273 sequence, thereby demonstrating that TCRs from different Vβ families (Vβ10b and Vβ6) interact with the same core region. Interestingly, hybridoma 20H5 was responsive to the 266–273 sequence. Although reactivity of hybridoma 20H5 was clearly affected by truncation of Phe265, a clear responsivity was seen, suggesting that the hybridoma needs few MHC-peptide complexes for activation. In conclusion, recognition of the 263–275 sequence by this set of DRB1*0401-restricted hybridomas is qualitatively different.

### Identification of MHC and TCR contact residues using four different T cell hybridomas

The fine specificity of the DRB1*0401-restricted hybridomas was analysed by the response to single site substituted analogues of the HC gp-39(263–275) sequence (Table 2). As expected, based on the binding data, substitution of the phenylalanine for alanine (F265A3, compound 12) showed a significant reduction in HLA-DR4 binding affinity (IC_50 _> 35). In contrast, all other single substituted APLs (compounds 10, 11, 13 to 22) bound HLA-DR4 with high relative affinity. Also, an APL with two alanine substitutions at positions 265 and 270 (compound 23) displayed a reduced affinity comparable to the A3 peptide. Interestingly, F265A3 was not recognized by both the 5G11 and 14G11 hybridomas. In contrast, reactivity of hybridoma 8B12 was affected to a lesser extent, whereas hybridoma 20H5 reactivity was only slightly affected. The data are in agreement with a lesser binding affinity of the F265A3 peptide. Although the four T-cell hybridomas differed in their fine specificity, some common features were detected. Peptides R263A1, S264A2, S270A8, V274A12 and G275A13 (Table 2, compounds 10, 11, 17, 21 and 22, respectively) did not affect the hybridoma response whereas substitutions at positions A268D6, S269A7, E271A9 and T272A10 (Table 2, compounds 15, 16, 18, 19, respectively) completely abrogated or significantly diminished the response of all four hybridomas, suggesting that these latter residues are primary TCR contacts. Secondary TCR contacts interfering with T-cell recognition of some, but not all, hybridomas were identified by substitutions at positions T266A4, L267A5 and G273A11 (compounds 13, 14, 20, respectively).

### Analysis of the polyclonal response to HC gp-39(263–275) *in vivo*

Previous studies had shown that the response to the HC gp-39 protein can be efficiently recalled by the immunodominant 263–275 epitope and visa versa [17]. In order to compare the cognate hybridoma data with a more physiological response *in vivo*, the HLA-DR4trg mice were immunized in the footpad with the WT peptide and, seven days later, the popliteal lymph node cells were assayed for their proliferative response and cytokine production when stimulated with the WT peptide or single site substituted APLs (compounds 12 to 20). A dose-dependent proliferative response to specific peptide (WT) was found (Table 3). In addition, we found significant, antigen-specific production of Mip1α (CCL3), RANTES (CCL5) and IFNγ in the supernatant of these cultures. In the polyclonal setting, the p1 anchor substituted F265A3 did not elicit a significant response. Furthermore, peptides A268D6, S269A7, E271A9 and T272A10 did not elicit a response, which suggests that the residues at these positions are essential for TCR interaction. Alternatively, clear activation was seen with both the L267A5 and S270A8 peptides, suggesting that these residues do not interact with the TCR. Interestingly, partial activation was seen with peptides T266A4 and G273A11. These data are in full agreement with the hybridoma data (except for L267A5). Comparable data were obtained with popliteal lymph node cells obtained from HC gp-39 (whole protein) immunized mice (data not shown).

**Table 3:** Antigen-specific response (proliferation and cytokine production) of popliteal lymph node cells from HC gp-39(263–275)-immunized huCD4, HLA-DR4 transgenic mice to single site substituted altered peptide ligands

					Concentration (pg/ml)		
					
Compound	HC gp39	Sequence	[Peptide] (μg/ml)	Prol (SI)	MIP1α	RANTES	IFNγ
1	263–275	RSFTLASSETGVG	10	**8**	**252**	**23**	**16**
			2	**6**	**128**	**13**	6
			0.4	**3**	25	6	<5
12	F265A3	RS**A**TLASSETGVG	10	**3**	27	9	<5
			2	1	<24	<6	<5
13	T266A4	RSF**A**LASSETGVG	10	**7**	**116**	**18**	**9**
			2	**4**	**47**	**11**	<5
14	L267A5	RSFT**A**ASSETGVG	10	**7**	**295**	**32**	**14**
			2	**6**	**112**	**11**	<5
15	A268D6	RSFTL**D**SSETGVG	10	**3**	29	<6	<5
			2	1	<24	<6	<5
16	S269A7	RSFTLA**A**SETGVG	10	2	<24	<6	<5
			2	2	<24	<6	<5
17	S270A8	RSFTLAS**A**ETGVG	10	**7**	**279**	**20**	**12**
			2	**5**	**86**	**12**	<5
18	E271A9	RSFTLASS**A**TGVG	10	1	<24	<6	<5
			2	1	<24	<6	<5
19	T272A10	RSFTLASSE**A**GVG	10	1	<24	<6	<5
			2	1	<24	<6	<5
20	G273A11	RSFTLASSET**A**VG	10	**4**	**47**	**15**	<5
			2	1	<24	<6	<5
		No peptide	0	1	<24	<6	<5

Antigen-specific responses (proliferation and cytokine production) were assessed at 96 h. Proliferation is expressed as stimulation index (SI; antigen specific cpm/background cpm). Background was 407 cpm. The standard deviation of measurements did not exceed 30%. For the proliferative assay an SI > 2 (bold) is regarded as positive. Cytokine production of Mip1α, RANTES and IFNγ is expressed in pg/ml. The limit of detection for these cytokines is 24, 5 and 6 pg/ml respectively. Values in bold are regarded as positive. Other cytokines assayed (such as IL-1α, IL-1β, IL-2, IL-3, IL-4, IL-5, IL-6, IL-10, IL-12p40, IL-12p70, IL-17, granulocyte colony-stimulating factor, granulocyte-macrophage colony-stimulating factor, tumor necrosis factor-α and cytokine-induced neutrophil chemoattractant (KC)) were not detected. Prol, proliferation.

In conclusion, the analysis of both the hybridoma panel and the polyclonal T cell response to the 263–275 epitope has led to the identification of MHC binding and TCR contact residues within this epitope. Phe265 was found to be the residue anchoring the peptide in the peptide binding groove, whereas positions Ala268, Ser269, Glu271 and Thr272 present the primary TCR contacts, affecting all specificities tested.

### Molecular modelling of HLA-DR4(B1*0401)/HC gp-39(263–275) complex

**Figure 3:**
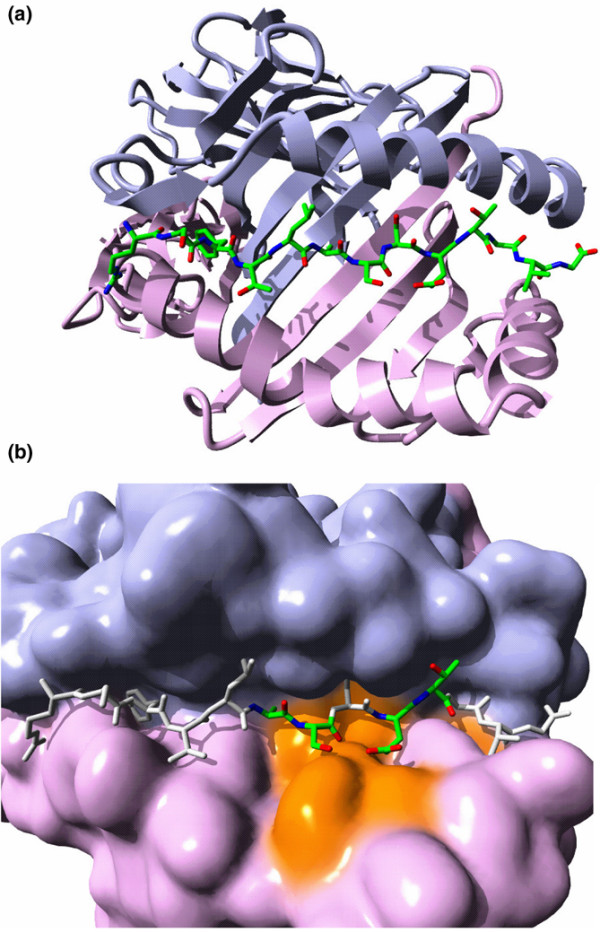
How T cells see the HC gp-39(263–275) epitope bound to HLA-DRB1*0401. **(a) **Structural model of the HLA-DR4(B1*0401)/HC gp-39(263–275) complex. Blue, α-chain; purple, β-chain. **(b) **Model of what the T-cell receptors 'see' when contacting the HLA-DR4(B1*0401)/HC gp-39(263–275) complex. A close up of the MHC surface representation is shown. Peptide residues are coloured by atom type if they are important in the contact, and grey if they are not (the sequence RS**F**TLASSETGVG is shown with the p1 anchor in bold; underlined residues are primary TCR contacts). MHC β-chain residues that might influence hybridoma activation are coloured orange. Molecular graphics created with YASARA [47] and PovRay [48].

The superposition of the published MHC-peptide-TCR complex crystal structures shows that the three-dimensional structures of all the MHC-peptide complexes are very similar, which implies that the structure of the HLA-DR4(B1*0401)/HC gp-39(263–275) complex can reliably be modelled. The position of the TCRs with respect to the MHC-peptide complex is more or less identical, but the orientation and, therefore, the specific contacts between the different TCRs and the MHC-peptide complexes differ. It is not possible, therefore, to predict the complete structure of a TCR/HLA-DR4(B1*0401)/HC gp-39(263–275) complex based on the available crystal structures. It is possible, however, to visualize what the TCR 'sees' when contacting the MHC-peptide complex. Figure 3a shows the final structural model of the HLA-DR4(B1*0401)/HC gp-39(263–275) complex. The average interaction energy between the MHC molecule and the peptide was calculated and compared to energies for the alanine substituted APL (Table 2). Good agreement was observed between the calculated average interaction energies and the experimentally observed binding strengths (data not shown), which validates the model.

Four TCR contact sites (Ala268, Ser269, Glu271 and Thr272) are important for polyclonal T cell activation. No information is available on important residues in the MHC α-chain, but for the β chain information on MHC restriction can be used (data not shown). The peptide does bind to HLA-DRB1*0402, B1*0405 and B1*0101 [7]. However, these complexes do not activate the B*0401-restricted hybridomas, so we conclude that residues differing in these β sequences might be important for activation. This leads to the view of the MHC-peptide complex in Figure 3b. Some of these MHC β-chain residues are the same as residues that are close to the TCR in the published crystal structure [38]. Other residues might influence the activation indirectly by modifying the positions of the peptide residues.

### MHC anchor substituted APL of HC gp-39(263–275)

A set of 12 non-classical APLs was made with side chain modifications at the primary MHC anchor residue (Figure 1). Interestingly, these modifications neither increased nor decreased the relative binding affinity as the novel APLs were found to bind HLA-DR4(B1*0401) with equally high affinity as the WT peptide (Figure 1). When analysed for T hybridoma reactivity, however, partial activation, as measured by impaired IL-2 production, was seen with peptide F265(3Tic) (compound 27) in all three T cell hybridomas (Figure 1). Peptide F265(Dip) (compound 28) led to partial activation in two out of three hybridomas (8B12 and 14G11), whereas peptide F265Hfe (compound 24) led to a reduced activation in one out of three (14G11) hybridomas.

**Figure 4:**
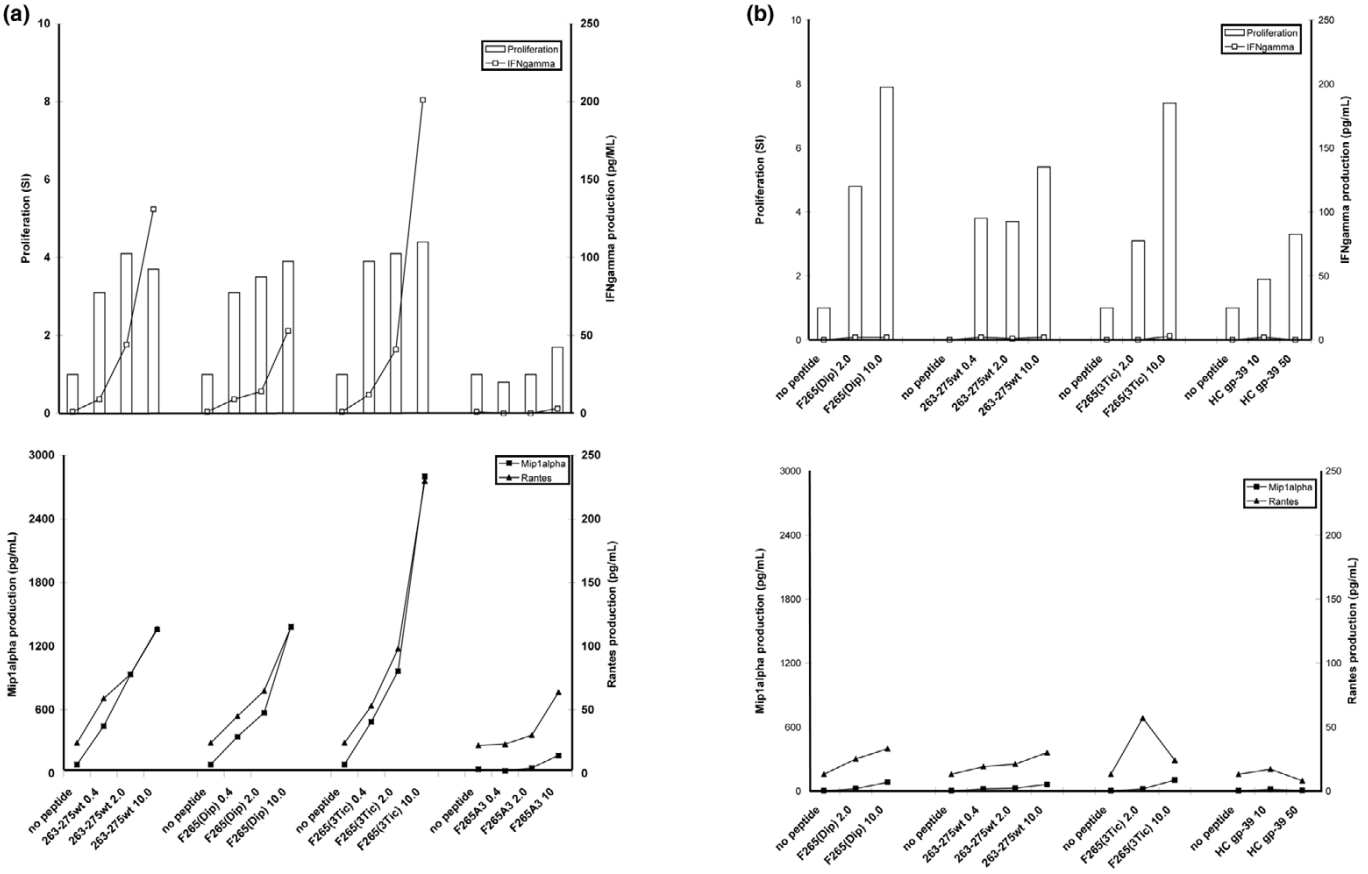
Response of polyclonal lymph node cells from HLA-DR4trg mice immunized with **(a) **wild-type (wt) peptide or **(b) **the F265(Dip) altered peptide ligand (APL). Antigen-specific response (proliferation, IFNγ production, upper panel; Mip1α and RANTES production, lower panel) of popliteal lymph node cells from HC gp-39(263–275)-immunized, HLA-DR4 transgenic mice to peptide (wild type and APLs) (a). Antigen-specific response (proliferation, IFNγ production, upper panel; Mip1α and RANTES production, lower panel) of popliteal lymph node cells from MHC variant F265(Dip)-immunized, HLA-DR4trg mice to peptide (F265(Dip), 263–275 wt, HC gp-39 and F265(3Tic)) (b).

Next, popliteal lymph node cells from WT peptide-immunized HLA-DR4trg mice were analysed for their *ex vivo *recall responses (proliferation and cytokine production) to the WT peptide, or the F265(3Tic) or F265(Dip) APLs. F265A3 (compound 12), with low affinity for HLA-DR4, served as a control. The HC gp-39(263–275) primed T cells showed comparable proliferative responses to the cognate peptide, and the F265(Dip) or F265(3Tic) APLs (Figure 4a). Interestingly, while the WT peptide elicited significant levels of IFNγ, Mip1α and RANTES, the F265(Dip) APL recalled lesser IFNγ production with unaltered Mip1α and RANTES production. In contrast, the F265(3Tic) APL recalled an increased production of these cytokines.

To further investigate the immune potential of the F265(Dip) APL, HLA-DR4trg mice were immunized with this variant. Popliteal lymph node cells of these mice showed a good proliferative activity when recalled *in vitro *with F265(Dip) (Figure 4b). The recall of the proliferative response was somewhat less with the 263–275 WT peptide or with HC gp-39 protein. Interestingly, the production of IFNγ, Mip1α and RANTES was barely detectable in the recall with F265(Dip). Strikingly, the recall with the WT peptide, the HC gp-39 protein or F265(3Tic) also failed to induce significant levels of these Th1 recruiting cytokines.

## Discussion

We and others have provided a strong case for a role of HC gp-39 as a target of the immune response in RA. The finding that the immunodominant epitope of HC gp-39 is endogenously presented by APCs in RA synovial tissue suggests that immunotherapeutic strategies based on the endogenous sequence may be beneficial for RA. In this study we identified an APL (F265(Dip)) with the capacity to skew the antigen-specific, pro-inflammatory response in HLA-DR4trg mice. The APL identified was not modified at the TCR contact sites but contains a modification at the key MHC anchor position. The latter quality may add to improved safety of APL therapy.

Ideally, the properties of an APL for immunotherapy of RA would blend: affinity for MHC class II binding; capabilities to engage polyclonal TCRs with sufficient avidity to alter pro-inflammatory function; a high resemblance to the naturally processed epitope; expression of the epitope at the site of chronic inflammation for engagement of auto-responsive T cells; and, recently, the prevention of unwanted immune reactivity [30,31].

In this study, we first characterized a panel of cognate T cell hybridomas. The four hybridomas were found to differ, as shown by different TCRVβ expression and/or fine epitope recognition. The heterogenous panel was used to identify the epitope residues interacting with MHC and/or the cognate TCRs using the single amino acid substitution approach. The core epitope contained one major residue (Phe265 at the p1 anchor position) important for binding to HLA-DR4. Given that we employed a peptide binding motif, imposing certain restrictions for the p1, p4, p6 and p9 positions, for the identification of this T cell epitope within HC gp-39 [7,41], some more impact of the single site substitutions at p4, p6 and p9 was anticipated. The observation that a dual site substitution (at p1 and p6) gave a similar low binding affinity as the p1 single substitution clearly underlines the importance of the p1 phenylalanine in this sequence as predicted based on the crystal structures [38,42]. Furthermore, four different amino acid positions within the core region were found to strongly interact with the TCRs since substitution did not affect HLA-DR4 binding but abrogated recognition in all four hybridomas. Notably, the interaction of position 268 with the TCR (A268D) was further substantiated using A268V6 and A268N6 as additional APLs (data not shown). The hybridoma data were confirmed when analysing the same peptides for recognition by polyclonal, cognate T cells from HLA-DR4trg mice, thereby showing that one APL may engage multiple clonalities.

Our analysis was extended to modifications at the MHC anchor residue. This strategy was inspired by the notion that unwanted immune reactivity as seen with APLs in clinical trials is thought to occur more vividly with classic APLs modified at the TCR contact sites, which may be 'more visible' to the immune system and may thus engage and activate novel clonalities [30,31]. We hypothesised that the p1 pocket, buried in the class II molecule, would allow for accommodation of modified side chains and, thus, selected non-natural amino acids with different side chain modifications not affecting the peptide backbone. Notably, these APLs displayed comparable affinities for binding HLA-DRB1*0401 as the WT peptide. Surprisingly, however, partial TCR agonists were identified using the T hybridoma panel. When assayed using polyclonal T cells from WT peptide-immunized HLA-DR4trg mice, the partial agonistic activity of F265(3Tic) was not confirmed. Instead, this APL showed an increased cytokine production. This heteroclitic response is explained by the notion that a conformational change in the peptide backbone is introduced by substitution with the more rigid bicyclic amino acid 3Tic which may give rise to additional clonalities. In contrast, the partial agonistic activity of the F265(Dip) APL was confirmed using WT peptide-sensitized lymph node cells from HLA-DR4trg mice. A lesser recall of IFNγ production, representative of a Th1 type of response, was observed.

In trials of APL in multiple sclerosis, increased reactivity to APLs and the native myelin basic protein were noted [31]. Thus, we investigated the immune potential of the F265(Dip) APL in the context of HLA-DR4. The results show that this APL does not give rise to increased immune responses to APL or native peptide/protein. Rather, the partial agonistic activity was confirmed by reduced cytokine production while maintaining antigen-specific proliferation. More importantly, neither the WT peptide nor the HC gp-39 protein was capable of recalling pro-inflammatory cytokine production (IFNγ, Mipα and RANTES). The data suggest that the F265(Dip) APL is redirecting the immune response to HC gp-39. Future experiments should address the capacity of this APL to modulate the HC gp-39-specific, HLA-DR4-restricted response in the context of experimental arthritis.

How to explain the immune modulating potential of this APL at the level of TCR recognition, triggering and beyond? The current paradigm is that the recognition of slightly altered ligands by T cells can lead to partial activation by interfering with the stability, clustering or duration of the TCR-mediated signal [43,44]. For the APLs identified here, it remains to be established whether effects on APL-MHC stability, density or duration of TCR activation can be reconciled with an inhibition of specific cytokine production in an otherwise productive response.

Although a link between RA and HC gp-39 has been established, the therapeutic efficacy of HC gp-39-based forms of therapy still await clinical proof [45,46].

## Conclusion

We have identified an APL resembling the endogenous epitope, which binds the RA-associated HLA-DRB1*0401 and is capable of dampening the polyclonal, pro-inflammatory cytokine response. Moreover, the apparent lack of priming novel reactivities suggests that this APL blends all the properties desired for evaluation in future clinical trials in RA.

## Abbreviations

APC = antigen-presenting cell; APL = altered peptide ligand; BLCL = B lymphoblastoid cell line; HC gp-39 = human cartilage glycoprotein-39; IFN = interferon; IL = interleukin; MHC = major histocompatibility complex; RA = rheumatoid arthritis; TCR = T-cell receptor; WT = wild type.

## Competing interests

Synthetic peptide modifications based on the endogenous RSFTLASSETGVG sequence of HC gp-39 are part of a patent application by NV Organon. The scientists responsible for the work described in the manuscript are employees of NV Organon (except for R Stenger), a pharmaceutical company. Upon acceptance, NV Organon will finance the article processing charge.

## Authors' contributions

AMHB, MJCvL, GFV, CMT, and CJvS designed the study. CMT and CJvS designed and synthesized the peptides. BWE and GFV performed the peptide-MHC binding studies. HH, LdHZ, RS, and CvD performed the hybridoma studies, the DR4trg studies and analysed the polyclonal T cell responses. MK was responsible for the peptide-MHC modelling studies. AMHB, MK, ESB and MJCvL prepared the manuscript. All authors read and approved the final manuscript.
